# Functional Characterization of a Root-Preferential and Stress-Inducible Promoter of Eca-miR482f in *Eucalyptus camaldulensis*

**DOI:** 10.3390/plants15010067

**Published:** 2025-12-25

**Authors:** Weihua Zhang, Qian Zhou, Xiaotong Wu, Shuyi Huang, Yuanzhen Lin

**Affiliations:** 1 Guangdong Academy of Forestry, Guangzhou 510520, China; zwh523@sinogaf.cn (W.Z.); 17704095209@163.com (S.H.); 2 Guangdong Provincial Key Laboratory of Silviculture, Protection and Utilization, Guangzhou 510520, China; 3 College of Forestry and Landscape Architecture, South China Agricultural University, Guangzhou 510642, China; xixi_buildshare@163.com (Q.Z.); wxt121387@163.com (X.W.)

**Keywords:** *Eucalyptus camaldulensis*, Eca-miR482f, root-preferential expression, stress inducibility

## Abstract

MicroRNAs (miRNAs) act as pivotal post-transcriptional regulators of gene expression in plant stress responses. However, the transcriptional regulation mechanisms governing miRNA genes themselves remain insufficiently characterized. This study focuses on Eca-miR482f, a previously identified cold-responsive miRNA from *Eucalyptus camaldulensis* that targets *EcaSIZ1*—a key component of the ICE1–CBFs–CORs cold signaling pathway. We first investigated the expression pattern of Eca-miR482f and found it exhibited root-preferential accumulation in *E. camaldulensis*. Under cold stress, it displayed divergent organ-specific responses: strong induction in roots and suppression in aerial tissues. To elucidate its transcriptional regulation, we cloned a 1938 bp promoter sequence upstream of the Eca-miR482f precursor. Bioinformatics analysis revealed that this promoter was highly conserved within the *Eucalyptus* genus and enriched with multiple cis-acting elements associated with stress responses—including a low-temperature-responsive element (LTR)—as well as hormone signaling, such as abscisic acid (ABA) and methyl jasmonate (MeJA)-responsive motifs. A series of 5′-deletion fragments were generated to delineate the functional regions within the promoter. Through transgenic approaches in both tobacco and *Arabidopsis*, we demonstrated that this promoter drove strong, root-preferential expression. Furthermore, it exhibited significant inducibility under cold and MeJA treatments. Systematic truncation analysis delineated specific promoter regions essential for maintaining this organ specificity and stress responsiveness, thus identifying potential functional modules. Briefly, our findings provide crucial insights into the transcriptional regulation of Eca-miR482f and uncover a valuable genetic tool for future biotechnological engineering of stress-tolerant woody plants via precise spatiotemporal modulation of gene expression.

## 1. Introduction

MicroRNAs (miRNAs) are small non-coding RNAs (approximately 17–25 nucleotides) that serve as crucial post-transcriptional regulators of gene expression in plants. By mediating either mRNA cleavage or translation inhibition, miRNAs participate in a wide range of biological processes, including development, metabolism, and stress responses [1]. Among the numerous miRNA families identified in plants, the miR482/2118 superfamily is evolutionarily conserved and functionally implicated in plant immunity, abiotic stress tolerance, and reproductive development [2]. For instance, in cotton (*Gossypium hirsutum*) [3] and tomato [4], miR482 family members targeted nucleotide-binding site-leucine-rich repeat (NBS-LRR) genes to fine-tune disease resistance pathways. In litchi (*Litchi chinensis*), miR482 triggered an amplifying regulatory cascade via two non-coding trans-acting genes (*LcTASL1* and *LcTASL2*), which generated phased small interfering RNAs (phasiRNAs) that cooperatively silence the gibberellin receptor gene *LcGID1*—suggesting a key role of this pathway in fruit and seed development [5]. In peanut (*Arachis hypogaea*), miR482-mediated regulation contributed to cold stress adaptation by modulating the expression of stress-responsive genes [6]. These studies underscore the evolutionary conservation and functional versatility of the miR482 family, which acts as a central hub integrating developmental programs and stress-responsive signaling through species-specific and context-dependent regulation of target genes. Despite extensive characterization of their post-transcriptional functions, the transcriptional regulatory mechanisms governing miR482 family expression remain poorly understood—particularly in woody plant species—limiting our ability to fully exploit its biological potential.

In plants, the expression of miRNA genes is governed by species-specific regulatory mechanisms, which are largely determined by their distinct promoter architectures [2]. As core regulatory modules, promoters enable precise spatiotemporal control of gene expression, and the functional dissection of their cis-acting elements is fundamental to deciphering transcriptional regulatory networks underlying diverse biological processes [7]. Previous bioinformatic analyses have identified numerous stress- and hormone-responsive cis-elements within miRNA promoters across different plant species. For instance, studies in rice (*Oryza sativa*) have revealed 16 environmental stress-associated promoter elements, including well-characterized cis-acting elements such as MYC, MYB, and WRKY71OS, as well as specific DNA domains and novel regulatory motifs [8]. Comparative analyses between *Arabidopsis* and rice further demonstrated species-specific cis-regulatory landscapes, with distinct sets of enriched elements identified in each species—providing critical insights into the molecular basis underlying differential miRNA expression patterns [9]. Additionally, ethylene-responsive miRNA promoters in rubber trees (*Hevea brasiliensis*) were found to contain various phytohormone-related cis-elements, suggesting the involvement of hormonal signaling pathways in the transcriptional regulation of miRNA [10]. In summary, these studies remain largely predictive, as most current knowledge on plant miRNA promoters is derived from bioinformatic predictions with limited experimental validation. Moreover, the cloning and functional characterization of miR482 promoters have not been reported to date, representing a major gap in our understanding of this conserved miRNA family’s regulatory hierarchy.

*Eucalyptus camaldulensis* is a widely cultivated fast-growing tree species with significant economic importance for pulp, paper, and bioenergy production. However, its productivity and geographical distribution are often constrained by abiotic stresses, particularly low-temperature extremes [11], highlighting the urgent need to dissect stress-responsive regulatory networks for breeding stress-resilient varieties. Our previous research identified 30 cold-responsive miRNAs in *E. camaldulensis* through high-throughput sequencing, with Eca-miR482f emerging as a key regulator targeting *EcaSIZ1*—a SUMO E3 ligase that modulated ICE1 stability through SUMOylation [11]. This finding positions Eca-miR482f as an important component in the cold response network, yet the transcriptional mechanisms governing its expression remain unexplored. To address these two knowledge gaps—i.e., the lack of functional validation of miR482 family promoters and the unclear transcriptional regulation of Eca-miR482f in *E. camaldulensis*—we systematically characterized the promoter of Eca-miR482f in this study. Our specific objectives were as follows: (1) to analyze the organ-specific expression pattern of Eca-miR482f and its transcriptional response to cold stress; (2) to clone the Eca-miR482f promoter and identify its cis-acting elements via bioinformatics analysis; (3) to validate the promoter activity of full-length and truncated fragments using transient expression in tobacco and stable transformation in *Arabidopsis*; and (4) to evaluate the responsiveness of the Eca-miR482f promoter to various abiotic stresses and phytohormonal treatments. Collectively, our results provide important insights into the transcriptional regulation of Eca-miR482f and lay a foundation for the utilization of this promoter in the genetic engineering of stress-tolerant plants.

## 2. Results

### 2.1. Expression Profiling of Eca-miR482f in E. camaldulensis

#### 2.1.1. Organ-Specific Expression of Eca-miR482f

**Figure 1:**
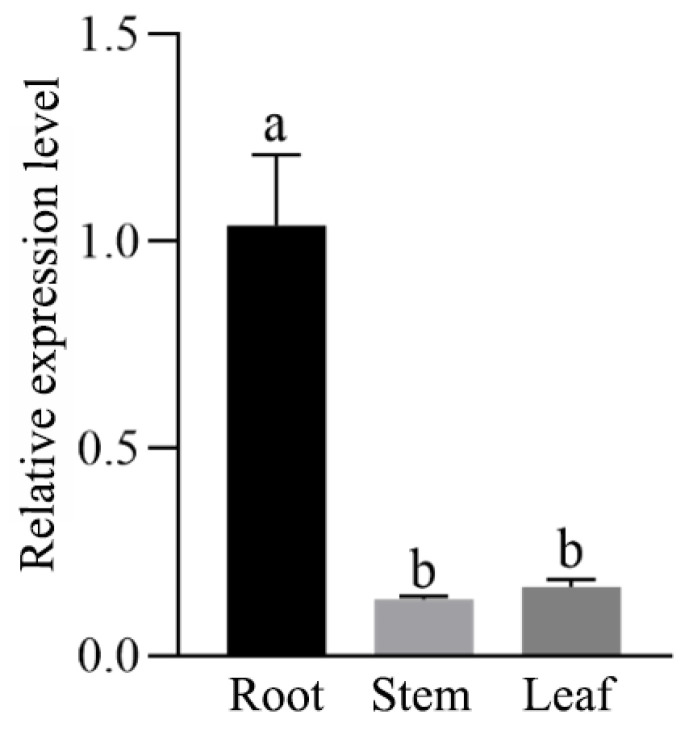
Organ-specific expression profile of Eca-miR482f in *E. camaldulensis*. Relative expression levels of Eca-miR482f in roots, stems, and leaves of 3-month-old tissue-cultured *E. camaldulensis* seedlings were quantified by qRT-PCR. *EcaRTEF* (Accession: AGC58243) was used as the internal reference gene for normalization. Note: Data are presented as mean ± standard deviation (SD) of three biological replicates. Different lowercase letters indicate statistically significant differences at α = 0.05 (one-way ANOVA followed by Duncan’s multiple range test).

The relative expression levels of Eca-miR482f in roots, stems, and leaves were quantified by qRT-PCR, with the *RTEF* gene of *E. camaldulensis* serving as the internal reference gene for normalization. As shown in Figure 1, Eca-miR482f was ubiquitously expressed across all test organs but exhibited significant organ-dependent variation. Specifically, transcript abundance in roots was markedly higher than in stems and leaves (*p* < 0.05). Although the expression level in leaves was slightly elevated compared to that in stems, the difference was not statistically significant. This root-preferential expression pattern suggests that Eca-miR482f may play a critical regulatory role in root development or physiology, potentially modulating nutrient uptake or stress resistance—key factors underlying the environmental adaptability of *E. camaldulensis*.

#### 2.1.2. Expression Response of Eca-miR482f to Cold Stress

**Figure 2:**
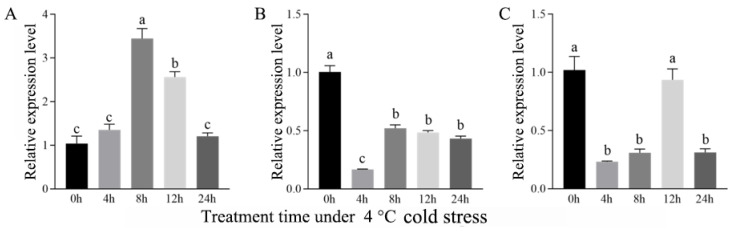
Organ-specific expression dynamics of Eca-miR482f under cold stress. Time-course expression analysis of Eca-miR482f in (**A**) roots, (**B**) stems, and (**C**) leaves of 3-month-old E. camaldulensis seedlings exposed to 4 °C cold stress. Samples were collected at 0, 4, 8, 12, and 24 h post-treatment. *EcaRTEF* (Accession: AGC58243) served as the internal reference gene for normalization. Note: Data are shown as mean ± SD (n = 3). Different lowercase letters denote significant differences at α = 0.05 (one-way ANOVA followed by Duncan’s multiple range test).

To explore the role of Eca-miR482f in cold tolerance, 3-month-old *E. camaldulensis* seedlings were exposed to 4 °C, and root, stem, and leaf samples were harvested at 0 h (control), 4 h, 8 h, 12 h, and 24 h post-treatment. qRT-PCR analysis revealed distinct organ-specific expression dynamics under cold stress (Figure 2). In roots, Eca-miR482f expression exhibited a transient “increase–decrease” pattern, peaking at 8 h (approximately 3.5-fold higher than the control, *p* < 0.05), followed by a gradual decline at 12 h and 24 h (Figure 2A). In stems, transcript levels decreased significantly at 4 h (*p* < 0.05), partially recovered by 8 h, but remained significantly below the control levels throughout the treatment period (*p* < 0.05), with no significant differences observed among the 8 h, 12 h, and 24 h time points (Figure 2B). In leaves, expression declined significantly at 4 h (*p* < 0.05), showed a slight transient increase at 12 h (comparable to the control level), and then decreased sharply again at 24 h (Figure 2C). These results demonstrate that Eca-miR482f mediates organ-specific responses to cold stress in *E. camaldulensis*, with roots displaying strong induction and aerial tissues showing general repression—reflecting divergent adaptive strategies across different plant organs.

### 2.2. Cloning and Sequence Characterization of the Eca-miR482f Promoter

#### 2.2.1. Cloning of the Eca-miR482f Promoter

Using specifically designed primers, the upstream regulatory region (putative promoter) of Eca-miR482f was amplified via PCR. PCR amplification yielded a single, specific band of approximately 1.9 kb, consistent with the expected size (Figure S1). The fragment was purified, cloned, and sequenced, confirming a total length of 1938 bp (designated as pEca-miR482f). Nucleotide composition analysis indicated that pEca-miR482f was AT-rich (56.91%), with thymine accounting for 32.25%—a hallmark of eukaryotic promoters that facilitates DNA unwinding during transcription initiation. BLAST sequence alignment revealed high sequence similarity between pEca-miR482f and homologous sequences from *E. camaldulensis* (97.12%) and *E. grandis* (88.63%) (Figure S2). These results confirm both the authenticity and evolutionary conservation of the cloned promoter, providing a reliable foundation for subsequent functional characterization.

#### 2.2.2. Bioinformatics Prediction of Cis-Acting Elements in the Eca-miR482f Promoter

**Figure 3:**
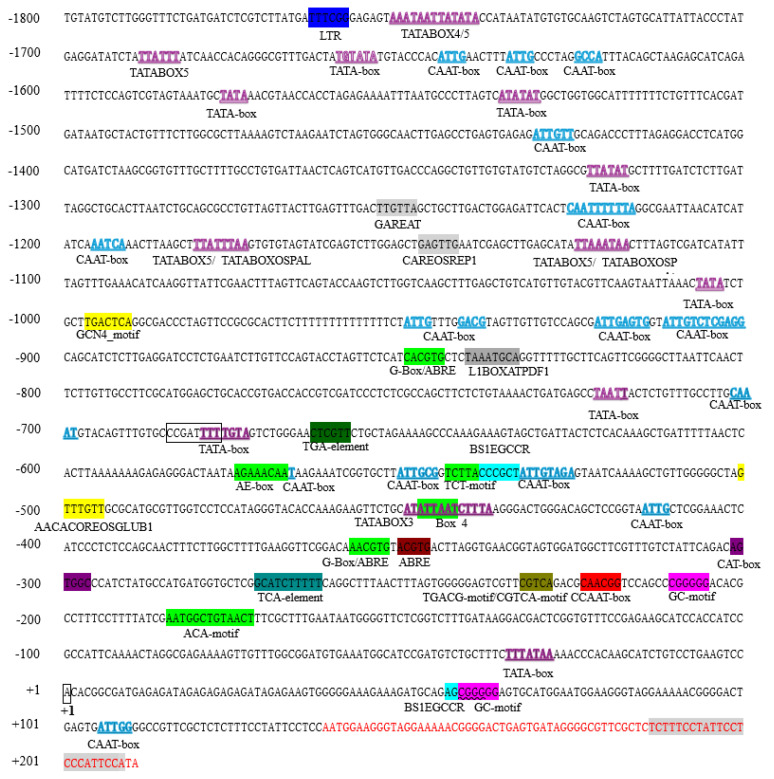
Schematic distribution of predicted cis-acting elements in the Eca-miR482f promoter. Note: Genomic organization of predicted cis-acting elements in the 1938 bp Eca-miR482f promoter (from –1800 bp to +210 bp relative to the transcription start site [+1], marked by a black box). Core elements: TATA-box (purple) and CAAT-box (blue). Stress/hormone-responsive elements: low-temperature-responsive element (LTR, red), abscisic acid-responsive element (ABRE, orange), methyl jasmonate-responsive motif (CGTCA-motif, green), and salicylic acid-responsive element (TCA-element, pink). Tissue-specific elements: endosperm-specific GCN4_motif (brown) and meristem-specific CAT-box (gray). Light-responsive elements: G-Box (cyan) and IBOXCORE (yellow). The precursor and mature sequences of Eca-miR482f are highlighted in red and gray, respectively.

To elucidate the regulatory functions of the Eca-miR482f promoter, cis-acting elements were predicted using the NewPlace and PlantCARE databases. The results are summarized in Tables S1 and S2, and Figure 3. A variety of cis-acting elements associated with stress responses, hormone signaling, and tissue-specific expression were identified, with partial overlap between the two databases. Specifically, the NewPlace database predicted one low-temperature response element (LTRE1HVBLT49), five light-responsive elements, five hormone-responsive elements (involving gibberellin, cytokinin, salicylic acid/auxin, and methyl jasmonate), and six tissue/development-related elements (including endosperm-, vascular-, primordium-, and pollen-specific motifs). PlantCARE identified one low-temperature response element (LTR) and one MYBHv1 binding site (CCAAT-box), five light-responsive elements, multiple hormone-responsive elements (ABRE for ABA, CGTCA- and TGACG-motif for MeJA, TCA-element for SA, TGA-element for auxin), and several tissue-specific elements (e.g., GAT-box for meristem, GC-motif for hypoxia, and GCN4-motif for endosperm). The distribution of these elements (Figure 3) underscores the complexity of the Eca-miR482f promoter, suggesting its capacity to integrate multiple environmental (e.g., cold, light) and endogenous (e.g., phytohormones) signals to fine-tune gene expression during growth and stress responses in *E. camaldulensis*.

### 2.3. Transient Expression Analysis of the Truncated Eca-miR482f Promoters in Tobacco

#### 2.3.1. LUC Fluorescence Activity Assay

**Figure 4:**
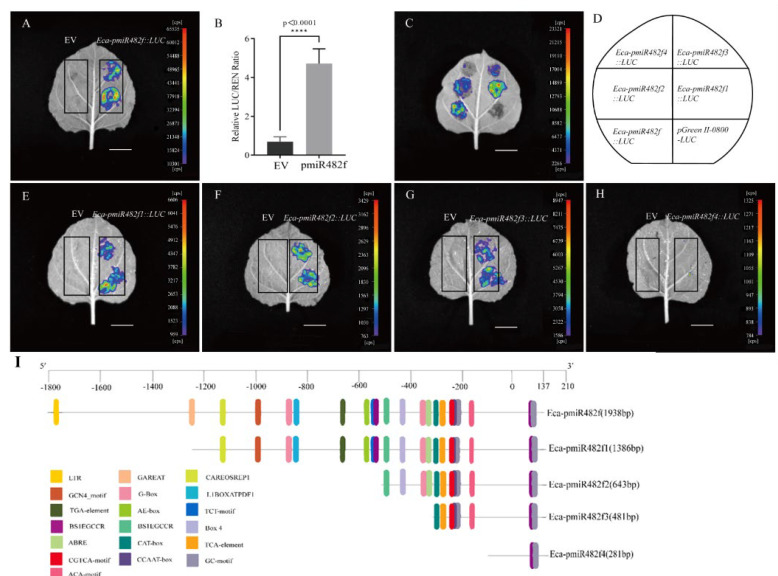
Transient LUC reporter assay of truncated Eca-miR482f promoters in tobacco. (**A**,**E**–**H**) In vivo LUC fluorescence imaging of tobacco leaves transiently transformed with LUC reporter constructs; black boxes indicate the infiltration area. (**B**) Quantitative analysis of LUC/REN activity ratios (mean ± SD, n = 3). (**C**,**D**) Fluorescence imaging of all constructs infiltrated into the same leaf to minimize leaf-to-leaf variation. (**I**) Schematic representation of 5’-truncated fragments of the Eca-miR482f promoter. Note: EV (empty pGreenII-0800-LUC vector, negative control); Eca-pmiR482f::LUC (full-length promoter); Eca-pmiR482f1::LUC to Eca-pmiR482f4::LUC (5’-truncated promoter fragments). The full-length promoter (Eca-pmiR482f) is 1938 bp. Truncated fragments are named based on their length (Eca-pmiR482f1: 1386 bp, Eca-pmiR482f2: 643 bp, Eca-pmiR482f3: 481 bp, Eca-pmiR482f4: 281 bp). Key cis-acting elements retained in each fragment are labeled as follows: LTR, low-temperature responsive; G-Box, light responsive; ABRE, ABA responsive; TATA/CAAT, core elements. Scale bars = 2 cm. **** denotes significant differences at *p* < 0.0001 (Student’s *t*-test vs. EV).

Transient expression assays were performed in tobacco leaves via *Agrobacterium tumefaciens*-mediated infiltration, with the empty pGreenII-0800-LUC vector (EV) used as a negative control. After 3 days of co-cultivation, LUC fluorescence was captured using an in vivo imaging system, and LUC/REN ratios were calculated to quantify promoter activity. As shown in Figure 4A,B, the full-length Eca-pmiR482f promoter drove strong LUC fluorescence, with a LUC/REN ratio significantly higher than that of the EV control (*p* < 0.0001, Student’s *t*-test). Among the truncated promoter variants (Figure 4E–H), Eca-pmiR482f1::LUC, Eca-pmiR482f2::LUC, and Eca-pmiR482f3::LUC exhibited moderate fluorescence and significantly higher LUC/REN ratios compared to EV (*p* < 0.05). In contrast, Eca-pmiR482f4::LUC showed only weak fluorescence, with a LUC/REN ratio slightly above that of EV but significantly lower than those of the other fragments (*p* < 0.05). To account for leaf-to-leaf variation, all constructs were infiltrated into the same leaf (Figure 4C,D), which confirmed that Eca-pmiR482f::LUC had the strongest activity, followed by Eca-pmiR482f1::LUC–Eca-pmiR482f3::LUC, while Eca-pmiR482f4::LUC exhibited the weakest activity. These results indicate that the core promoter region resides within the 281 bp Eca-pmiR482f4 fragment, whereas the upstream regions (−1800 to −1310 bp) contain enhancer elements that substantially enhance transcriptional activity.

#### 2.3.2. GUS Histochemical Staining

**Figure 5:**
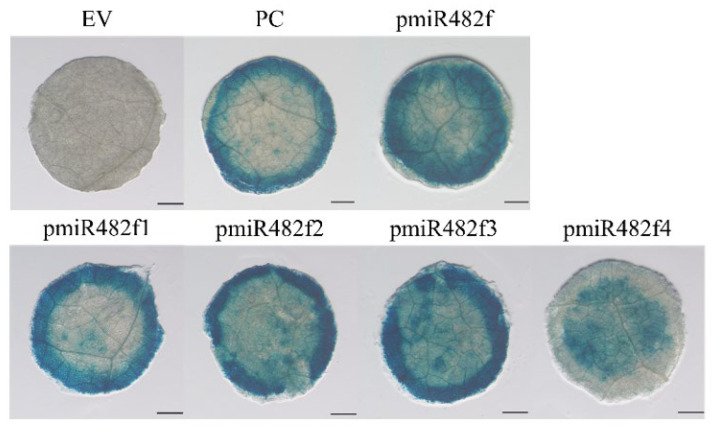
GUS histochemical staining of tobacco leaves transiently transformed with the truncated Eca-miR482f promoters. Note: EV (empty pHGWFS.7 vector, negative control); PC (CaMV35S::GUS, positive control); Eca-pmiR482f::GUS (full-length promoter); Eca-pmiR482f1::GUS to Eca-pmiR482f4::GUS (truncated promoter constructs). Leaves were stained 3 days post-infiltration and decolorized with 70% ethanol. Scale bars = 1 mm.

GUS histochemical staining was performed on tobacco leaves transiently transformed with pHGWFS.7-derived vectors. The empty pHGWFS.7 vector (EV) was used as a negative control, and the CaMV35S::GUS construct served as a positive control. After 3 days of co-cultivation, the infiltrated leaves were subjected to GUS staining. As shown in Figure 5, the EV control exhibited no detectable blue staining, whereas the CaMV35S::GUS positive control displayed intense blue staining. All Eca-miR482f promoter constructs (full-length and truncated variants) successfully directed GUS expression: Eca-pmiR482f::GUS, Eca-pmiR482f1::GUS, Eca-pmiR482f2::GUS, and Eca-pmiR482f3::GUS showed strong blue staining, with intensities comparable to or exceeding that of the positive control. In contrast, Eca-pmiR482f4::GUS exhibited only weak blue staining. These results are consistent with the LUC activity data from the transient expression assay, further confirming that the upstream regions beyond the 281 bp core fragment contain positive regulatory elements that enhance the transcriptional activity of the Eca-miR482f promoter.

#### 2.3.3. GUS Activity Under Abiotic Stress and Exogenous Hormone Treatments

**Figure 6:**
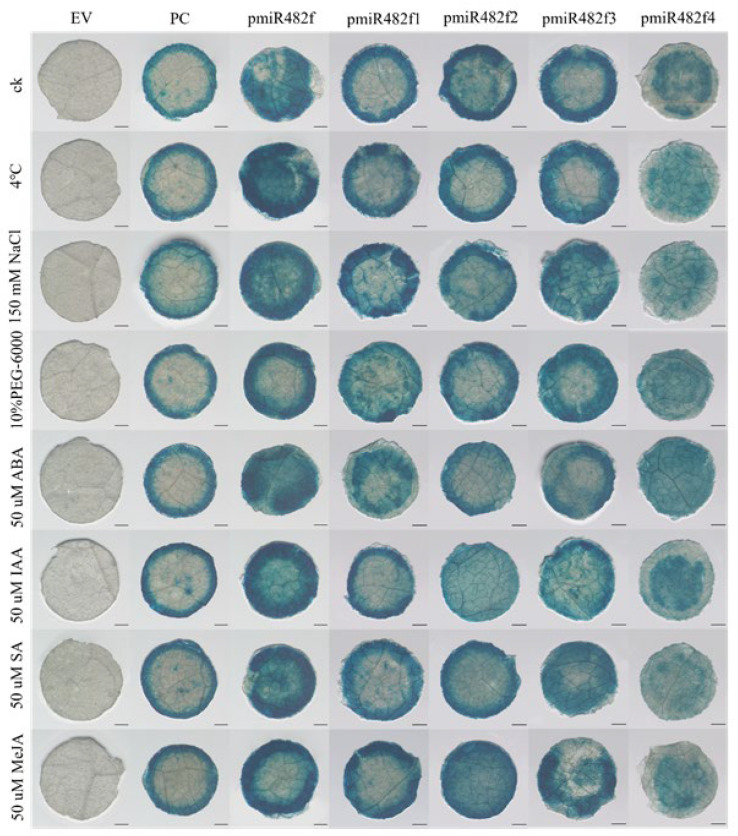
GUS histochemical staining of transiently transformed tobacco leaves under abiotic stress and exogenous hormone treatments. Note: GUS histochemical staining of transiently transformed *N. benthamiana* leaves subjected to 24 h treatments: CK (distilled water, control), 4 °C (cold stress), 150 mM NaCl (salt stress), 10% (*w*/*v*) PEG-6000 (drought stress), 50 μM abscisic acid (ABA), 50 μM indole-3-acetic acid (IAA), 50 μM salicylic acid (SA), and 50 μM methyl jasmonate (MeJA). EV (empty pHGWFS.7 vector), PC (CaMV35S::GUS), Eca-pmiR482f::GUS, and truncated promoter lines (Eca-pmiR482f1::GUS to Eca-pmiR482f4::GUS). Scale bars = 1 mm.

**Figure 7:**
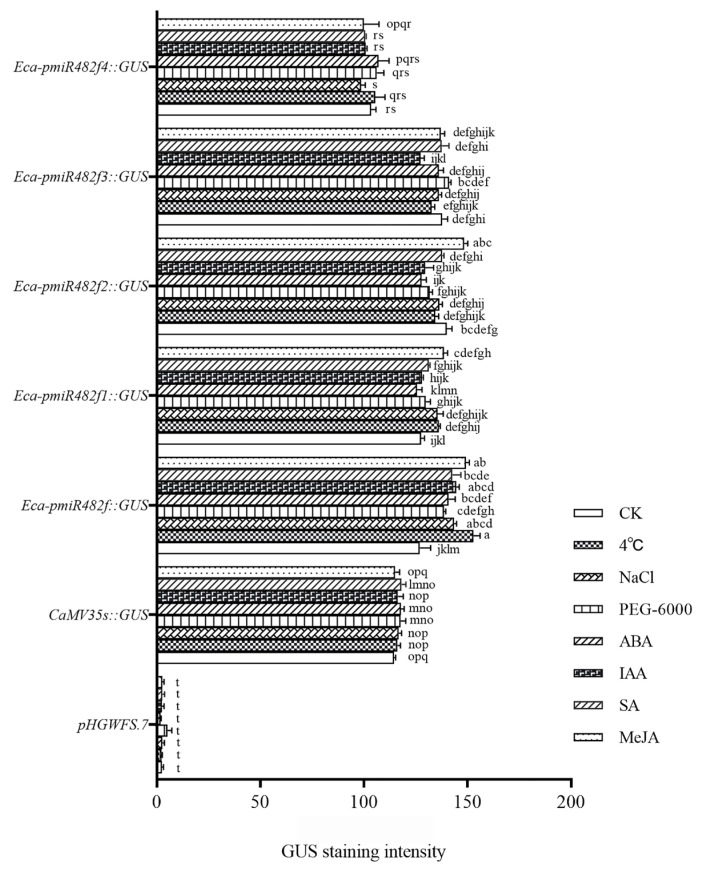
Quantitative analysis of GUS staining intensity under abiotic stress and exogenous hormone treatments. Note: Quantification of GUS staining intensity was performed using ImageJ software 1.52a. Data are presented as mean ± SD (n = 5). Different lowercase letters indicate significant differences among groups at α = 0.05 (two-way ANOVA followed by Duncan’s multiple range test).

To assess the stress and hormone responsiveness, transiently transformed tobacco leaves were subjected to abiotic stresses (4 °C for cold, 150 mM NaCl for salt, and 10% PEG-6000 for drought) and phytohormone treatments (50 μM ABA, IAA, SA, and MeJA), with distilled water used as the control (CK). After 24 h of treatment, GUS staining intensity was quantified using ImageJ (Figure 6 and Figure 7). The EV control exhibited no detectable GUS staining across all treatments (GUS intensity ≈ 0), while the CaMV35S::GUS positive control showed uniform, high-level staining (intensity ≈ 110) with no significant differences among treatments. The full-length Eca-pmiR482f::GUS construct exhibited significantly higher GUS staining intensity under all treatments compared to the control (CK) (*p* < 0.05), with the strongest induction observed under 4 °C cold stress (≈180) and MeJA treatment (≈150). The truncated promoter variants exhibited divergent responsiveness patterns: Eca-pmiR482f1::GUS responded significantly to 4 °C, NaCl, and MeJA (*p* < 0.05), but showed no significant response to ABA, IAA, or PEG; Eca-pmiR482f2::GUS exhibited significant responsiveness only to MeJA (*p* < 0.05); Eca-pmiR482f3::GUS and Eca-pmiR482f4::GUS showed no significant differences in GUS intensity between any treatment and the CK. Regarding the lack of significant induction by NaCl, PEG, ABA, IAA, and SA, further optimization of treatment concentrations and durations may be required to elicit a detectable response. These findings suggest that the cold-responsive element (LTR) resides within the −1800 and −1310 bp, the MeJA-responsive element is localized between −1310 and −505 bp, and the core 281 bp region lacks stress- or hormone-responsive cis-acting elements.

### 2.4. Stable Expression Analysis of the Eca-miR482f Promoter in Arabidopsis

#### 2.4.1. Tissue-Specific and Developmental Expression of the Eca-miR482f Promoter

**Figure 8:**
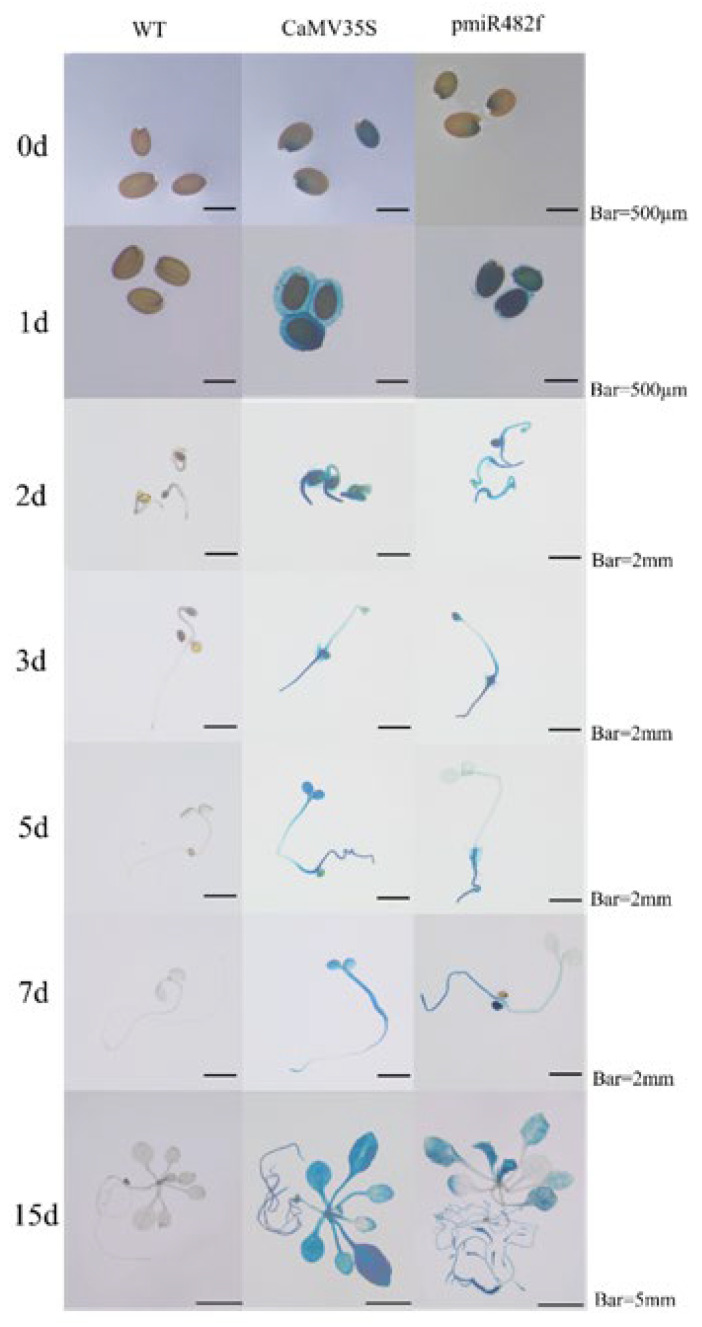
GUS staining of Eca-pmiR482f::GUS transgenic *Arabidopsis* at early developmental stages. GUS histochemical staining of T3 transgenic *Arabidopsis thaliana* (Columbia-0 ecotype) expressing Eca-pmiR482f::GUS at 0–15 days post-germination (dpg). Note: WT (wild-type Arabidopsis, negative control); CaMV35S::GUS (positive control). Scale bars: 500 μm (0–1 dpg), 2 mm (2–7 dpg), 5 mm (15 dpg).

**Figure 9:**
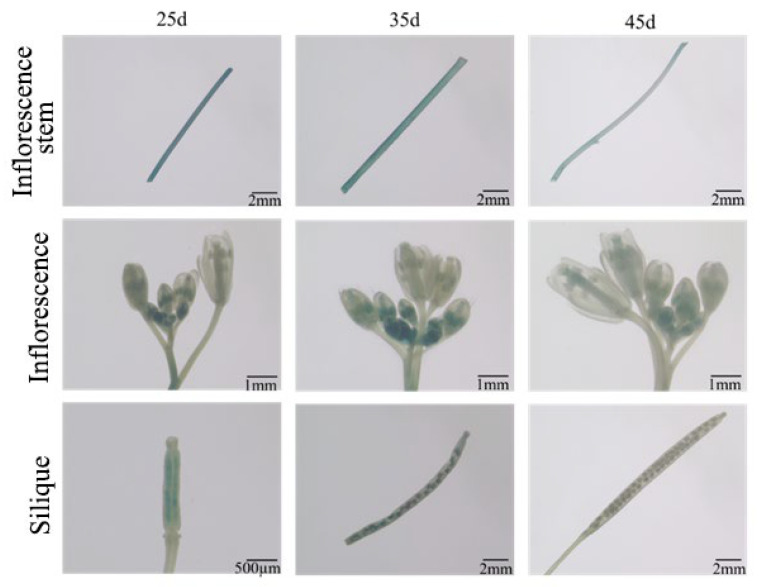
GUS staining of Eca-pmiR482f::GUS transgenic *Arabidopsis* at late developmental stages. GUS histochemical staining of T_3_ Eca-pmiR482f::GUS transgenic Arabidopsis at 25 days (vegetative stage), 35 days (reproductive stage), and 45 days (seed maturation stage). Shown are inflorescence stems, inflorescences, and siliques. Note: Scale bars: 500 μm (silique), 1 mm (inflorescence), 2 mm (inflorescence stem).

**Figure 10:**
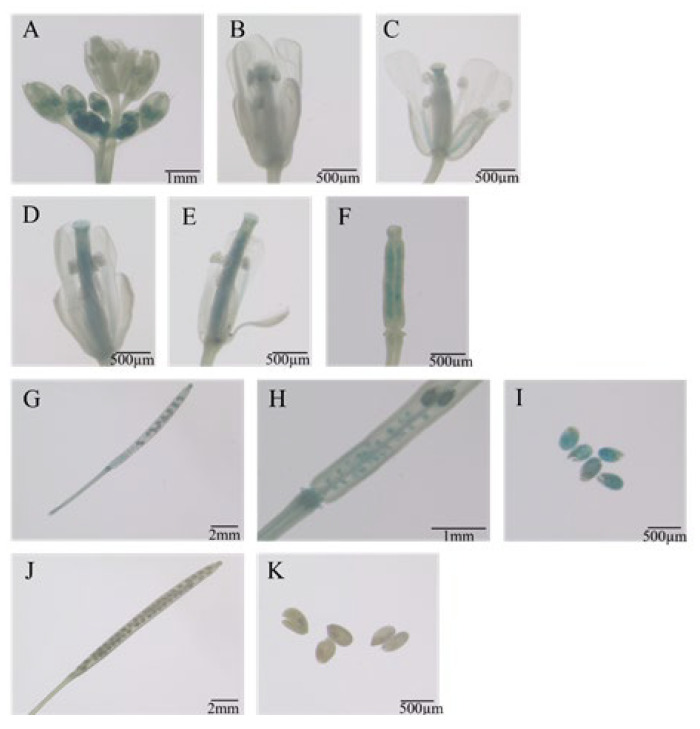
GUS staining of floral tissues and siliques in transgenic *Arabidopsis* with Eca-pmiR482f::GUS: (**A**) inflorescence; (**B**) flower at anthesis; (**C**) senescent flower; (**D**,**F**) fertilization stage; (**G**) immature silique; (**H**) ovule; (**I**) immature seed; (**J**) mature silique; (**K**) mature seed. Note: Samples in panels (**A**–**I**) and (**J**,**K**) were collected from 15-day-old and 45-day-old T_3_ transgenic plants, respectively. Scale bars are indicated in the figure.

**Figure 11:**
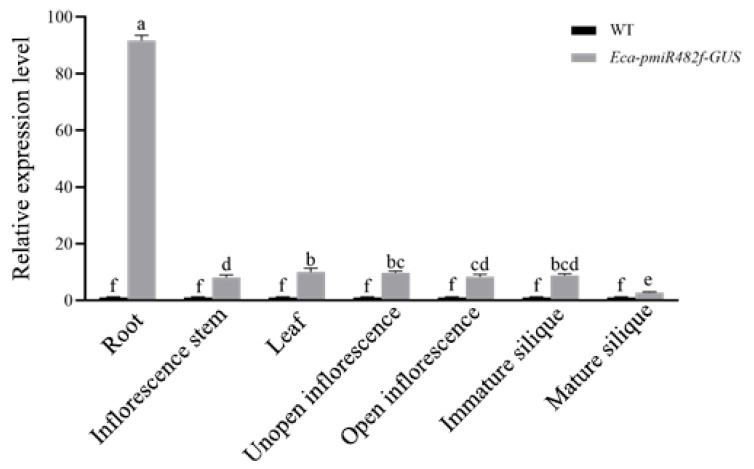
Tissue-specific GUS expression in 45-day-old transgenic *Arabidopsis*. qRT-PCR analysis of GUS transcript levels in different tissues of T_3_ Eca-pmiR482f::GUS transgenic *Arabidopsis*. *AtActin* (Accession:AT3G46520) was used as the internal reference gene for normalization. Note: Data are presented as mean ± SD (n = 3). Different lowercase letters indicate significant differences at α = 0.05 (two-way ANOVA followed by Duncan’s multiple range test).

GUS histochemical staining was performed on T_3_ transgenic *Arabidopsis* plants at different developmental stages: 0–15 days (germination/seedling stage), 25 days (vegetative stage), 35 days (reproductive stage), and 45 days (seed maturation stage). Wild-type (WT) *Arabidopsis* and CaMV35S::GUS transgenic plants served as negative and positive controls, respectively. During early development (0–15 days), GUS expression in Eca-pmiR482f::GUS seeds was first detected at the hilum at 1 day post-germination (dpg), with the GUS staining spreading to roots and cotyledons over time (Figure 8). By 7 dpg, strong GUS staining was observed in roots (especially root tips) and newly emerging true leaves, while cotyledon staining gradually faded. At 15 dpg, staining was most intense in roots and young leaves. During the vegetative, reproductive, and seed maturation stages (Figure 9), GUS staining progressively weakened in inflorescence stems. Unopened floral buds exhibited stronger staining in pistils and stamens compared to opened flowers. Following pollination, GUS activity re-emerged in ovaries, with strong signals in ovules, but was absent in mature siliques. More specifically, GUS expression was robust in early floral buds, ovarian ovules, and immature seeds, but became weak or undetectable in late-stage flowers and mature siliques (Figure 10). qRT-PCR analysis of 45-day-old transgenic plants (Figure 11) showed that GUS expression was significantly highest in roots (*p* < 0.05), followed by unopened buds and immature siliques, with the lowest expression detected in mature siliques and leaves. These results demonstrate that the Eca-miR482f promoter drives spatiotemporally specific expression in *Arabidopsis*–exhibiting root-preferential expression in seedlings and dynamically regulated expression during reproductive development—paralleling its organ-specific expression in *E. camaldulensis* and suggesting evolutionary functional conservation of its regulatory mechanism.

#### 2.4.2. Stress Responsiveness of the Eca-miR482f Promoter

**Figure 12:**
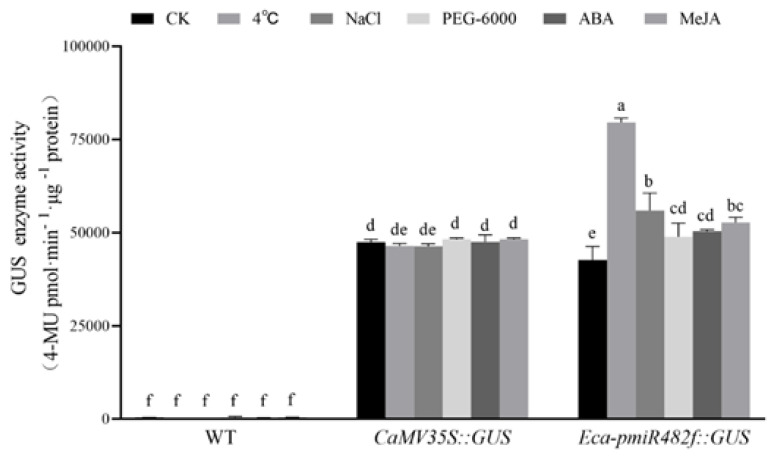
Quantitative GUS enzyme activity in transgenic *Arabidopsis* with Eca-pmiR482f::GUS under stress and hormone treatments. Fluorometric quantification of GUS enzyme activity in 15-day-old T_3_ Eca-pmiR482f::GUS transgenic *Arabidopsis* seedlings subjected to 24 h treatments. GUS activity was measured using 4-methylumbelliferyl-β-D-glucuronide (4-MUG) as the substrate. Note: WT (wild-type *Arabidopsis*, negative control); CaMV35S::GUS (positive control). Data are shown as mean ± SD (n = 3). Different lowercase letters denote significant differences at α = 0.05 (two-way ANOVA followed by Duncan’s multiple range test).

To confirm the stress responsiveness of the Eca-miR482f promoter in a stable transgenic system, 15-day-old T_3_ Eca-pmiR482f::GUS seedlings were subjected to the same abiotic stress and phytohormone treatments as those used in tobacco transient assay. GUS activity was quantified via fluorometric assay (Figure 12). WT exhibited no detectable GUS activity, while the CaMV35S::GUS positive control maintained stable activity across all treatments (*p* > 0.05 vs. CK). In Eca-miR482f::GUS transgenic seedlings, GUS activity was significantly induced by cold stress (1.6-fold higher than that of CK, *p* < 0.05), with the induced levels exceeding those of the CaMV35S::GUS control. No significant induction of GUS activity was observed under NaCl, PEG, ABA, or MeJA treatments. These results confirm that the Eca-miR482f promoter is cold-inducible in *Arabidopsis*, consistent with the findings from the tobacco transient assays and the presence of the LTR element identified in the promoter sequence.

#### 2.4.3. Driving Activity of Truncated Eca-miR482f Promoter in Transgenic Arabidopsis

*(1)*  *GUS Histochemical Staining*

**Figure 13:**
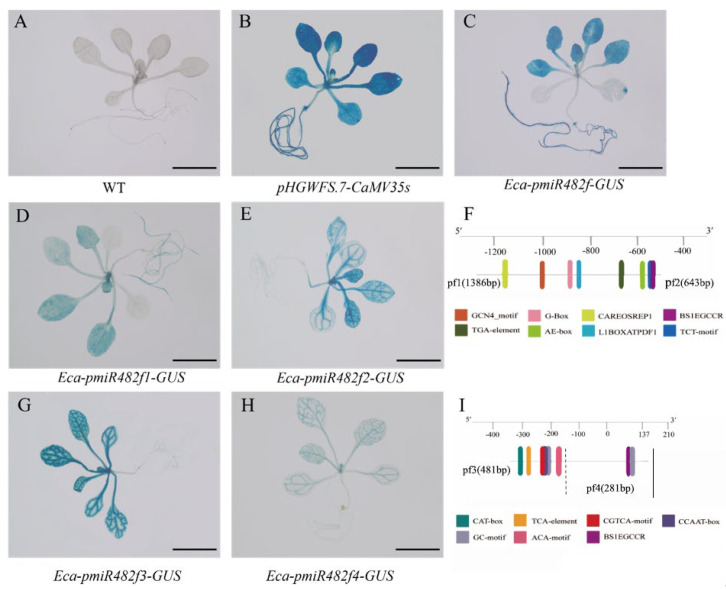
GUS staining of transgenic *Arabidopsis* with truncated Eca-miR482f promoters. (**A**) WT (wild-type, negative control); (**B**) CaMV35S::GUS (positive control); (**C**) Eca-pmiR482f::GUS (full-length promoter); (**D**,**E**,**G**,**H**) Eca-pmiR482f1::GUS to Eca-pmiR482f4::GUS (truncated promoter lines); (**F**,**I**) Schematic diagrams of key cis-acting elements lost between truncations (blue = root-specific elements, red = leaf-specific enhancer elements). Note: Scale bars = 5 mm.

GUS histochemical staining of 15-day-old T_3_ transgenic *Arabidopsis* harboring truncated promoters revealed distinct differences in promoter activity and tissue specificity (Figure 13). The Eca-pmiR482f1::GUS construct drove GUS expression in both roots and leaves, although the staining intensity was weaker than that of the full-length promoter. In contrast, Eca-pmiR482f2::GUS expression was restricted to leaves, with no detectable staining in roots. Eca-pmiR482f3::GUS exhibited only weak GUS staining in leaves, while Eca-pmiR482f4::GUS activity was nearly undetectable, similar to the WT control. These results suggest that the root-specific regulatory element resides within the −1310 to −505 bp region (a segment lost in the Eca-pmiR482f2 truncation), leaf-specific enhancer elements are localized between −505 to −343bp (a region absent in Eca-pmiR482f3), and the core promoter region alone (−143 to +138 bp, corresponding to Eca-pmiR482f4) is insufficient to drive detectable expression in *Arabidopsis*.

*(2)*  *qRT-PCR Analysis of GUS Expression*

**Figure 14:**
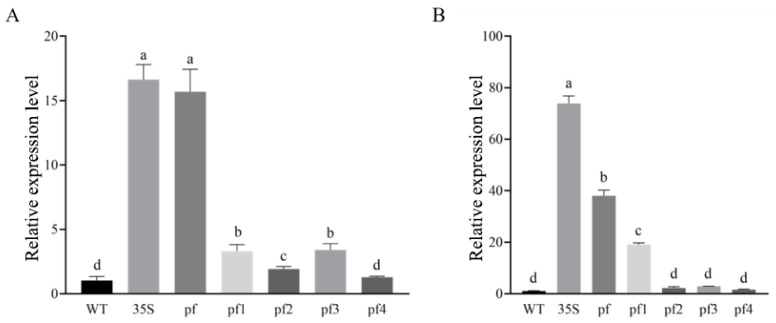
qRT-PCR validation of GUS expression in truncated promoter transgenic *Arabidopsis* lines. Relative GUS expression levels in (**A**) leaves and (**B**) roots of 15-day-old T_3_ transgenic *Arabidopsis* harboring truncated Eca-pmiR482f promoters. Note: WT (wild-type); 35S (CaMV35S::GUS, positive control); pf (Eca-pmiR482f::GUS, full-length promoter); pf1–pf4 (Eca-pmiR482f1::GUS to Eca-pmiR482f4::GUS, truncated lines). *AtActin* (Accession:AT3G46520) was used as the internal reference gene for normalization. Data are presented as mean ± SD (n = 3). Different lowercase letters indicate significant differences at α = 0.05 (one-way ANOVA followed by Duncan’s multiple range test).

qRT-PCR analysis was performed on leaves and roots from 15-day-old seedlings to validate the GUS staining patterns (Figure 14). In leaves, the full-length Eca-pmiR482f::GUS construct exhibited the highest GUS expression level (*p* < 0.05), followed by Eca-pmiR482f1::GUS–Eca-pmiR482f3::GUS, while Eca-pmiR482f4::GUS expression was comparable to that of the WT control. In roots, significant GUS expression was detected exclusively in seedlings harboring the full-length Eca-pmiR482f::GUS and Eca-pmiR482f1::GUS constructs (*p* < 0.05); no detectable expression was observed in Eca-pmiR482f2::GUS–Eca-pmiR482f4::GUS lines. These findings further refine the delineation of functional regions within the Eca-pmiR482 promoter: the −1310 to −505 bp region is essential for root specificity, the −505 to −143 bp region functions as a leaf-specific enhancer, and the −143 to +138 bp core region confers basal promoter activity—albeit insufficient to drive detectable expression independently.

## 3. Discussion

### 3.1. Functional Implications of Eca-miR482f Expression Patterns

In the present study, Eca-miR482f was found to be ubiquitously expressed in roots, stems, and leaves of *E. camaldulensis*, with significantly higher transcript levels in roots compared to stems and leaves (Figure 1). This root-preferential expression pattern is consistent with previous reports on miR482a in *Pinus densata* [12] and miR482 in mulberry [13], suggesting a conserved regulatory role of miR482 family members in root physiology. Our previous high-throughput small RNA sequencing study indicated that Eca-miR482f may participate in the ICE1–CBFs–CORs cold signaling pathway in *E. camaldulensis* [11], and its direct cleavage of the EcaSIZ1 transcript was confirmed by 5’-RNA ligase-mediated rapid amplification of cDNA ends (5’-RLM-RACE) [14]. Consistent with the typical post-transcriptional repressive role of miRNAs [15], the observed downregulation of Eca-miR482f in stems and leaves under 4 °C cold stress suggests that it likely represses EcaSIZ1 expression through RNA silencing mechanisms, such as transcript cleavage or translational inhibition. This regulatory mode is analogous to the function of ahy-miR482 in peanut, which enhances cold tolerance by suppressing *AhWDRL* expression [5]. Conversely, overexpression of miR482b in tomato compromises resistance to *Bacillus cinerea* [16], underscoring the functional divergence among miR482 homologs across plant species. The concomitant upregulation of *EcaSIZ1* following Eca-miR482f downregulation in stems and leaves of *E. camaldulensis* under cold stress further supports the involvement of the Eca-miR482f-EcaSIZ1 regulatory module in cold response.

Notably, Eca-miR482f exhibited distinct organ-specific expression dynamics under cold stress: a transient “increase-decrease” pattern in roots (peaking at 8 h with a 3.5-fold induction) versus downregulation in stems and leaves (Figure 2). Such tissue-dependent miRNA expression profiles are commonly observed in plants; for instance, miR156a is downregulated in leaves but upregulated in roots of *Medicago sativa* under drought stress [17], and miR396 family members show opposite expression patterns in leaves and roots of soybean under salt stress [18]. These findings suggest that Eca-miR482f may mediate tissue-specific roles in *E. camaldulensis* cold adaptation—potentially enhancing stress tolerance in roots while repressing energy-consuming processes in aerial tissues to prioritize resource allocation toward stress defense.

### 3.2. Cis-Acting Elements of the Eca-miR482f Promoter

The 1938 bp Eca-miR482f promoter cloned in this study exhibits high AT content (56.91%), a typical feature of plant promoters that facilitates DNA unwinding during transcription initiation [19]. Sequence alignment revealed 97.12% similarity with the *E. camaldulensis* genome and 88.63% similarity with *E. grandis* (Figure S2), reflecting evolutionary conservation within the *Eucalyptus* genus while retaining species-specific variations [20]. Similar AT-rich regions have been functionally implicated in promoter activity; for example, the *Arabidopsis PIP2;8* promoter contains an AT-rich segment bound by the PLATZ4 transcription factor [21], supporting the functional relevance of AT enrichment in the Eca-miR482f promoter.

Bioinformatic prediction using the NewPlace and PlantCARE databases identified a variety of cis-acting elements, including core promoter elements (TATA-box, CAAT-box) and stress/hormone-responsive elements (LTR, ABRE, MeJA-responsive motifs) (Tables S1 and S2). The consistent prediction of key elements—such as LTR (involved in cold response) and GCN4-motif (associated with endosperm-specific expression)—across both tools enhances the reliability of the predictions and their functional relevance. For instance, LTR elements have been demonstrated to mediate cold responsiveness in promoters of *PpcERF5* [22] and *VaERF095* [23], consistent with the cold-induced activity of the Eca-miR482f promoter observed in our functional assays.

Moreover, complementary predictions between NewPlace and PlantCARE underscore the value of integrated bioinformatic approaches: NewPlace identified endosperm-specific and hypoxia-responsive elements not detected by PlantCARE, whereas PlantCARE uncovered meristem-specific elements absent in NewPlace. Such complementarity has also been reported in promoter studies of *Panicum virgatum* profilin [24] and *Rosa multiflora* U6 genes [25], highlighting the importance of multi-tool validation for comprehensive cis-acting element identification. Variations in element sequences (e.g., MeJA-responsive T/GBOXATPIN2 in NewPlace versus CGTCA/TGACG-motifs in PlantCARE) may reflect differences in transcription factor binding specificities, enabling the Eca-miR482f promoter to integrate multiple signaling pathways for fine-tuned gene expression.

### 3.3. Functional Validation of the Eca-miR482f Promoter

Transient expression in tobacco and stable transformation in *Arabidopsis* confirmed the robust transcriptional activity of the Eca-miR482f promoter (Figure 4 and Figure 8), consistent with previous reports on the *Prunus sibirica PsMATE40* promoter [26]. The spatio-temporal expression pattern of the Eca-miR482f promoter in *Arabidopsis* (Figure 8, Figure 9 and Figure 10)—characterized by root-preferential expression in seedlings and dynamically regulated expression during reproductive development—correlates with the distribution of predicted cis-acting elements. For example, the endosperm-specific GCN4-motif is likely responsible for driving promoter activity during seed germination, as observed in the promoters of peanut *8A4R19G1* [27] and rice *GluB-1* [28]. Additionally, hormone-responsive elements (ABRE, TGA-element) may contribute to the promoter’s activity in ovules and immature siliques, given the well-established roles of auxin in *Arabidopsis* ovule development [29] and ABA in cotton fiber initiation [30].

The Eca-miR482f promoter exhibited cold-inducible activity, with a 1.6-fold increase in GUS expression under 4 °C stress (Figure 7), validating the functional relevance of the LTR element identified in its sequence. This result mirrors the cold-responsive characteristics of the cotton *GhDREB1* promoter [31]. Although ABRE elements (typically associated with responses to salt, drought, and ABA) were predicted in the Eca-miR482f promoter, only mild, non-significant induction was observed under these treatments. The lack of significant induction by ABA, despite the presence of ABRE elements, may be due to the absence of necessary co-factors in our experimental system or insensitivity to the current experimental conditions, which renders them non-functional in the absence of additional stress cues. This discrepancy may reflect species-specific regulatory mechanisms or the requirement for synergistic interactions among multiple elements—consistent with observations in *Jatropha curcas JcGASA6*, where SA/ABA/GA3-responsive elements did not confer the expected hormone induction [32]. In contrast, MeJA treatment significantly enhanced the transcriptional activity of the Eca-miR482f promoter, which aligns with reports on the *Panax quinquefolius Pq3-O-UGT2* [33] and *Gossypium barbadense GbCHI* promoters [34], underscoring the promoter’s responsiveness to jasmonate signaling pathways.

### 3.4. Modular Functional Regions of the Eca-miR482f Promoter Governing Organ Specificity

Truncation analysis revealed a positive correlation between promoter fragment length and transcriptional activity: the full-length promoter exhibited the highest activity, while the shortest fragment (281 bp, Eca-pmiR482f4) showed minimal transcriptional activity (Figure 13). This length-dependent activity pattern is attributed to the progressive loss of core promoter elements (TATA-box and CAAT-box)—cis-acting motifs essential for transcription initiation efficiency [35,36]. A similar length-dependent activity pattern has been reported for the *Medicago truncatula Dof32* promoter [37], highlighting the evolutionary conservation of this promoter structural feature. Furthermore, the reduced activity of Eca-pmiR482f1 relative to the full-length promoter suggests the presence of transcriptional repressive elements within the −1800 to −1310 bp region—a regulatory phenomenon also observed in the *Neolamarckia cadamba NcSUT1* promoter [38], indicating a common modular regulatory strategy among plant promoters.

Organ-specific expression analysis of truncated promoter variants further identified distinct functional regulatory modules within the Eca-miR482f promoter. Specifically, the −1310 to −505 bp region harbors root-specific regulatory elements (e.g., L1BOXATPDF1), as Eca-pmiR482f2, Eca-pmiR482f3, and Eca-pmiR482f4—all lacking this genomic segment—failed to drive detectable root expression (Figure 13). L1BOXATPDF1 has been implicated in root hair development [39] and root tissue differentiation [40], providing direct evidence supporting its role in mediating root-preferential expression. Additionally, the −505 to −343 bp region is inferred to harbor leaf-specific enhancer elements, as Eca-pmiR482f3 (lacking this segment) exhibited significantly reduced leaf transcriptional activity compared to longer promoter fragments. These results demonstrate that the Eca-miR482f promoter comprises discrete modular regions that cooperatively fine-tune tissue-specific expression patterns and stress-responsive transcriptional activity in a combinatorial manner.

## 4. Materials and Methods

### 4.1. Plant Materials and Growth Conditions

Three-month-old tissue-cultured plantlets of *E. camaldulensis* clone CV103 were prepared as previously described [11]. Tobacco (*Nicotiana benthamiana)* and *Arabidopsis thaliana* (Columbia-0 ecotype) were cultivated in a greenhouse under controlled environmental conditions: 25 °C, 16 h light/8 h dark photoperiod, 60% relative humidity, and a light intensity of 200 μmol·m^−2^·s^−1^.

### 4.2. RNA Extraction and Quantitative Real-Time PCR (qRT-PCR)

For organ-specific expression analysis, root, stem, and leaf samples were collected from 3-month-old *E. camaldulensis* plantlets. For cold stress treatment, plantlets were transferred to a 4 °C growth chamber, and samples were collected at 0, 4, 8, 12, and 24 h post-treatment. Total RNA was extracted using a Column Plant Total RNA Extraction Kit (Sangon, Shanghai, China), and genomic DNA contamination was removed using an RNase-Free DNA Erasing Kit (Sangon, Shanghai, China).

Given the short length of miRNAs (17–25 nt), stem-loop reverse transcription (RT) was used for cDNA synthesis [41]. qRT-PCR was performed on a Light Cycler 480 system (Roche, Switzerland) using TB Green Premix Ex Taq II (TaKaRa, Dalian, China). *EcaRTEF* (Accession: AGC58243) was used as the internal reference gene for *E. camaldulensis* [42], and *Atactin* (Accession:AT3G46520) served as the reference gene for *A. thaliana*. The relative expression levels were calculated using the 2^−ΔΔCT^ method [43]. All primers used in this study are listed in Table S3.

### 4.3. Cloning and Sequence Analysis of the Eca-miR482f Promoter

Genomic DNA was isolated from *E. camaldulensis* leaves using an Ezup Column Super Plant Genomic DNA Extraction Kit (Sangon, Shanghai, China). Based on the 2000 bp upstream sequence of miR482f from *E. camaldulensis* and *E. grandis* genomes (https://www.ncbi.nlm.nih.gov/datasets/genome/GCF_016545825.1, accessed on 3 August 2024), specific primers (pmiR482f-F/R) were designed using DNAMAN 9.0 (Table S4). PCR amplification was carried out using ApexHF HS DNA Polymerase (Aikerui, Changsha, China) under the following conditions: initial denaturation at 94 °C for 30 s; 35 cycles of 94 °C for 10 s, annealing at 55 °C for 5 s, and extension at 72 °C for 2 min; and a final extension at 72 °C for 5 min.

The PCR product was purified using a SanPrep Column DNA Gel Extraction Kit (Sangon, Shanghai, China), cloned into the pTOPO vector (Zhongmei Taihe, Beijing, China), and sequenced by Sangon. The cloned promoter sequence was aligned with the genomic sequences of *E. camaldulensis* and *E. grandis* using DNAMAN 9.0. Cis-acting elements were predicted using PlantCARE (https://bioinformatics.psb.ugent.be/webtools/plantcare/html/, accessed on 2 April 2025) and NewPLACE (https://www.dna.affrc.go.jp/PLACE/?action=newplace, accessed on 2 April 2025) databases, and the distribution of elements was visualized using TBtools software 2.025 [44].

### 4.4. Construction of Promoter Reporter Vectors

Based on the distribution of predicted cis-acting elements, four 5’-truncated promoter fragments (1386 bp, 643 bp, 481 bp, 281 bp; Figure 4I) were designed and amplified using specific primers with homologous arms (Table S5). The full-length and truncated promoter fragments were individually inserted into the pGreen II-0800-LUC vector (for LUC reporter assays) and pHGWFS.7 vector (for GUS reporter assays) via homologous recombination and Gateway^®^ cloning technology, respectively.

The recombinant vectors were transformed into *Escherichia coli* DH5α competent cells, and positive clones were verified by Sanger sequencing. The correct plasmids were subsequently introduced into *Agrobacterium tumefaciens* strain GV3101 via the freeze–thaw method, and positive agrobacterial clones were confirmed by PCR amplification and sequencing.

### 4.5. Transient Expression Assays in Tobacco

*Agrobacterium* strains harboring LUC or GUS reporter vectors were cultured in LB medium supplemented with 100 μg·mL^−1^ kanamycin and 30 μg·mL^−1^ rifampicin at 28 °C with shaking at 200 rpm until OD_600_ reached 0.6. The bacterial suspension was centrifuged at 5000 rpm for 10 min, resuspended in infiltration buffer (10 mM MgCl_2_, 10 mM MES, and 200 μM acetosyringone), and incubated at room temperature for 2–3 h in the dark.

For LUC activity assays, the *Agrobacterium* suspension was injected into the abaxial side of 4–5-week-old tobacco leaves. After 3 days of co-cultivation, 1 mM D-luciferin was sprayed onto the leaves, and LUC fluorescence was detected using a NightSHADE LB985 plant in vivo imaging system (Berthold, Bad Wildbad, Germany). LUC and Renilla luciferase (REN) activities were quantified using a Dual-Luciferase Reporter Assay Kit II (Biyuntian, Shanghai, China), with REN serving as the internal reference to normalize LUC activity.

For GUS activity assays, the agrobacterial suspension was infiltrated into tobacco leaves, and GUS histochemical staining was performed after 3 days of co-cultivation using a GUS Staining Kit (Kulibo, Beijing, China). Stained leaves were decolorized with 70% ethanol (*v*/*v*), and staining intensity was quantified using ImageJ software [45].

For stress and hormone treatments, transiently transformed tobacco plants were subjected to 4 °C cold stress, irrigation with 150 mM NaCl (salt stress) or 10% (*w*/*v*) PEG-6000 (drought stress), or foliar spraying with 50 μM abscisic acid (ABA), indole-3-acetic acid (IAA), salicylic acid (SA), or methyl jasmonate (MeJA). Distilled water was used as the control (CK). GUS staining and activity quantification were conducted after 12 h of treatment.

### 4.6. Stable Transformation Assays in Arabidopsis

The recombinant pHGWFS.7 vectors were transformed into *Arabidopsis* via the floral dip method [46]. T_0_ seeds were surface-sterilized with 25% (*v*/*v*) NaClO and 0.1% (*v*/*v*) Triton X-100 for 10 min, rinsed three times with sterile distilled water, and sown on MS medium supplemented with 25 μg·mL^−1^ hygromycin and 50 μg·mL^−1^ timentin. The seeds were germinated and grown at 22 °C with a 16 h/8 h light/dark photoperiod. Resistant seedlings (T_1_ generation) were transplanted to soil and self-pollinated to obtain T_3_ homozygous lines.

For GUS histochemical staining, T_3_ transgenic *Arabidopsis* plants at different growth stages (0–15-day seedlings, 25-day vegetative plants, 35-day reproductive plants, and 45-day seed-maturing plants) were collected and immersed with X-Gluc solution (5-bromo-4-chloro-3-indolyl-β-D-glucuronic acid). Stained tissues were decolorized with 70% (*v*/*v*) ethanol and observed under a SZX16 stereomicroscope (Olympus, Tokyo, Japan).

For quantitative GUS activity assays, total protein was extracted from transgenic *Arabidopsis* tissues using GUS Extraction Buffer (Kulibo, Beijing, China). GUS activity was measured using a GUS Gene Quantitative Detection Kit (Kulibo, Beijing, China) with 4-MUG as the substrate. Fluorescence intensity was detected using a Vaioskan LUX microplate reader (Thermo Scientific, Waltham, MA, USA) at excitation and emission wavelengths of 365 nm and 456 nm, respectively.

### 4.7. Data Statistical Analysis

All experiments were performed with three biological replicates. Data were analyzed using R software 4.2.0. Differences among groups were assessed by one-way or two-way analysis of variance (ANOVA) followed by Duncan’s multiple range test. Statistical significance was defined as *p* < 0.05. Graphs were generated using GraphPad Prism 9.5.

## 5. Conclusions

This study systematically characterized the expression profile of Eca-miR482f and the functional properties of its promoter in *E. camaldulensis*. Key findings are summarized as follows: (1) Eca-miR482f exhibited root-preferential expression and organ-specific responsiveness to cold stress, potentially modulating cold tolerance via the Eca-miR482f-EcaSIZ1 module; (2) the cloned 1938 bp Eca-miR482f promoter was evolutionarily conserved among *Eucalyptus* species, featuring AT-rich regions and a diverse repertoire of cis-acting elements associated with stress responses, hormone signaling and organ-specific regulation; (3) the Eca-miR482f promoter drove robust spatiotemporal expression in tobacco and Arabidopsis, with specific responsiveness to cold stress and MeJA; and (4) systematic truncation analysis delineated discrete modular regions within the Eca-miR482f promoter that governed transcriptional strength and organ specificity. These results provide novel insights into the transcriptional regulation of Eca-miR482f and its functional role in mediating stress adaptation, laying a foundation for the genetic improvement of cold tolerance in *Eucalyptus* via precision molecular breeding strategies.

## Data Availability

The original contributions presented in this study are included in the article/Supplementary Material. Further inquiries can be directed to the corresponding author.

## Supplementary Materials

The following supporting information can be downloaded at: https://www.mdpi.com/article/10.3390/plants15010067/s1, Table S1: Cis-acting elements predicted in the Eca-miR482f promoter using the NewPlace database; Table S2: Cis-acting elements predicted in the Eca-miR482f promoter using the PlantCARE database; Table S3: Primers used for qRT-PCR. Table S4: Primers used for Eca-miR482f promoter cloning; Table S5: Primers used for promoter vector construction; Table S6: Abbreviations used in the study; Figure S1: Agarose gel electrophoresis of the PCR-amplified Eca-miR482f promoter fragment; Figure S2: Sequence alignment of the cloned Eca-miR482f promoter (pmiR482f) with homologous sequences from *E. camaldulensis* (ec-pmiR482f) and *E. grandis* (eg-pmiR482f).
