# Supplementary material for: Functional Characterization of a Root-Preferential and Stress-Inducible Promoter of Eca-miR482f in Eucalyptus camaldulensis
Source: Plants (Basel). 2025 Dec 25;15(1):67. doi: 10.3390/plants15010067 (PMC12787873; doi:10.3390/plants15010067)
Supplement: Supplementary file 1 [file plants-15-00067-s001.zip › plants-4036031-supplementary.pdf]

Supporting Information for Zhang's manuscript

Table S1. Cis-acting elements predicted in the Eca-miR482f promoter using the NewPlace database

Element Name	Core Sequence	Number	Functional Annotation
AACACOREOSGLUB1	AACAAAC	1	Endosperm-specific expression enhancer
ACGTOSGLUB1	GTACGTG	1	Endosperm-specific expression regulator
GCN4OSGLUB1	TGAGTCA	1	Endosperm-specific expression element
IBOXCORE	GATAA	4	Light-responsive element
SORLIP1AT	GCCAC	2	Light response element
SORLIP2AT	GGGCC	2	Light-responsive element
SORLIP5AT	GAGTGAG	1	Light-responsive element
CACGTGMOTIF	CACGTG	1	Light-responsive element
CAREOSREP1	CAACTC	1	Gibberellin-responsive repressor element
GAREAT	TAACAAR	1	Gibberellin-responsive enhancer element
CPBCSPOR	TATTAG	1	Cytokinin-responsive element
ASF1MOTIFCAMV	TGACG	1	Salicylic acid/auxin-responsive element
T/GBOXATPIN2	AACGTG	1	Methyl jasmonate-responsive element
BS1EGCCR	AGCGGG	2	Vascular tissue-specific expression element
L1BOXATPDF1	TAAATGYA	1	Epidermal primordium -specific element
POLLEN1LELAT52	AGAAA	14	Pollen-specific expression element
LTRE1HVBLT49	CCGAAA	1	Low-temperature-responsive element
CAATBOX1	CAAT	12	Core regulatory element
TATABOX3	TATTAAT	1	Core regulatory element
TATABOX4	TATATAA	1	Core promoter element
TATABOX5	TTATTT	4	Core promoter element
TATABOXOSPAL	TATTTAA	2	Core promoter element

Table S2. Cis-acting elements predicted in the Eca-miR482f promoter using the PlantCARE database

Element Name	Sequence	Number	Functional Annotation
ACA-motif	AATTACAGCCATT	1	Light-responsive element
AE-box	AGAAACAA	1	Light-responsive element
Box 4	ATTAAT	1	Light-responsive element
TCT-motif	TCTTAC	1	Light-responsive element
G-Box	CACGTG	2	Light-responsive element
ABRE	ACGTG	4	Abscisic acid (ABA)-responsive element
TCA-element	CCATCTTTTT	1	Salicylic acid (SA)-responsive element
CGTCA-motif	CGTCA	1	Methyl jasmonate (MeJA)-responsive element
TGACG-motif	TGACG	1	Methyl jasmonate (MeJA)-responsive element
TGA-element	AACGAC	1	Auxin-responsive element
LTR	CCGAAA	1	Low-temperature-responsive element
GC-motif	CCCCCG	2	Hypoxia-specific responsive element
CCAAT-box	CAACGG	1	MYBHv1 binding site
CAT-box	GCCACT	1	Meristem-specific expression element
GCN4_motif	TGAGTCA	1	Endosperm-specific expression element
TATA-box	TATATA	13	Core promoter element
CAAT-box	CAAAT	14	Core regulatory element

Table S3. Primers used for qRT-PCR

Primer	Primer sequence (5'→3')	Target gene	Species
Eca-miR482f-F	GCGCGTCTTTCCTATTCCTC	Eca-miR482f	*E. camaldulensis*
miRNAs-R	GTGCAGGGTCCGAGGT	Universal miRNA reverse primer	—
EcaRTEF-F	TCCAATCCGAGTCGCTGTCATTGT	EcaRTEF (reference)	*E. camaldulensis*
EcaRTEF-R	TGATGAGCCTCTCTGGTTTGACC	EcaRTEF (reference)	*E. camaldulensis*
AtActin-F	CTTCGTCTTCCACTTCAG	Atactin (reference)	*A. thaliana*
AtActin-R	ATCATACCAGTCTCAACAC	Atactin (reference)	*A. thaliana*
GUS-F	GCACGGGAATATTTCGCCAC	GUS	*A. thaliana*
GUS-R	ATAACGGTTCAGGCACAGCA	GUS	*A. thaliana*

Table S4. Primers used for Eca-miR482f promoter cloning

Primer	Primer sequence (5'→3')
pmiR482f-F	TGTATGTCTTGGGTTTCTGATG
pmiR482f-R	GGAGGAATAGGAAAGAGAGCG

Table S5. Primers used for promoter vector construction

Primer	Primer sequence (5'→3')	Vector
LUC-pmiR482f-F	ctatagggcgaattgggtaccTGTATGTCTTGGGTTTCTGATGATC	pGreen II-0800-LUC
LUC-pmiR482f1-F	ctatagggcgaattgggtaccGCTGCTTGACTGGAGATT	pGreen II-0800-LUC
LUC-pmiR482f2-F	ctatagggcgaattgggtaccGCTAGTTTGTTGCGCATGCGT	pGreen II-0800-LUC
LUC-pmiR482f3-F	ctatagggcgaattgggtaccTAGGTGAACGGTAGTGGA	pGreen II-0800-LUC
LUC-pmiR482f4-F	ctatagggcgaattgggtaccTTGATAAGGACGACTCGGT	pGreen II-0800-LUC
LUC-pmiR482f-R	cgctctagaactagtggatccGGAGGAATAGGAAAGAGAGCGAA	pGreen II-0800-LUC
GUS-pmiR482f-F	ggggacaagtttgtacaaaaaagcaggctTGTATGTCTTGGGTTTCTGATGATC	pHGWFS.7
GUS-pmiR482f1-F	ggggacaagtttgtacaaaaaagcaggctGCTGCTTGACTGGAGATT	pHGWFS.7
GUS-pmiR482f2-F	ggggacaagtttgtacaaaaaagcaggctGCTAGTTTGTTGCGCATGCGT	pHGWFS.7
GUS-pmiR482f3-F	ggggacaagtttgtacaaaaaagcaggctTAGGTGAACGGTAGTGGA	pHGWFS.7
GUS-pmiR482f4-F	ggggacaagtttgtacaaaaaagcaggctTTGATAAGGACGACTCGGT	pHGWFS.7
GUS-pmiR482f-R	ggggaccactttgtacaagaaagctgggtGGAGGAATAGGAAAGAGAGCGAA	pHGWFS.7

Note: Lowercase letters indicate vector homologous arms.

Table S6. Abbreviations used in the study

Abbreviation	Full name
miRNA	MicroRNA
phasiRNAs	phased small interfering RNAs
ABA	abscisic acid
MeJA	methyl jasmonate
SA	salicylic acid
IAA	indole-3-acetic acid
NBS-LRR	nucleotide-binding site-leucine-rich repeat
LTR	low-temperature-responsive element
WT	Wild-type
EV	empty vector
CK	control
GUS	β-glucuronidase
LUC	luciferase
REN	Renilla luciferase
dpg	day post-germination
4-MUG	4-methylumbelliferyl-β-D-glucuronide
qRT-PCR	Quantitative Real-Time PCR
5'-RLM-RACE	5'-RNA ligase-mediated rapid amplification of cDNA ends
RT	reverse transcription
ANOVA	analysis of variance

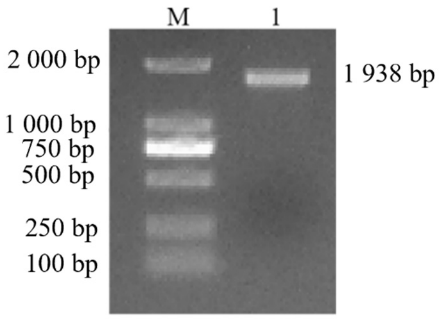

Figure S1. Agarose gel electrophoresis of the PCR-amplified Eca-miR482f promoter fragment. Note: M: 2000 bp DNA ladder; Lane 1: PCR product of the Eca-miR482f promoter.

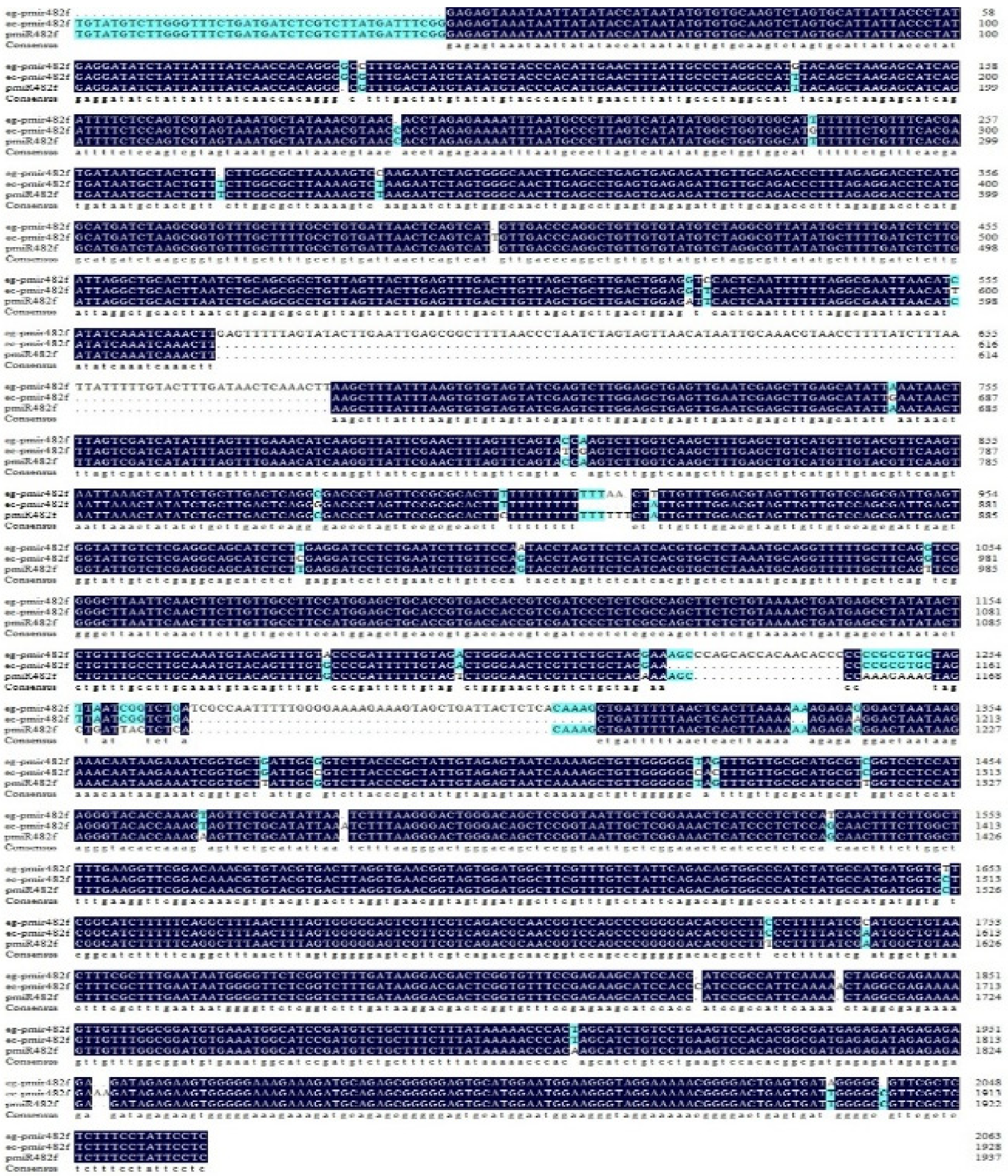

Figure S2. Sequence alignment of the cloned Eca-miR482f promoter (pmiR482f) with homologous sequences from *E. camaldulensis* (ec-pmiR482f) and *E. grandis* (eg-pmiR482f). Note: Alignment was performed using DNAMAN 9.0. Identical nucleotides are shaded in black, while similar nucleotides are shaded in gray. Gaps ("-") were introduced to optimize alignment.
